# A Rapid RT-RAA Assay for Visual Detection of Ebola Virus: Advancing Early Diagnosis in Resource-Limited Settings

**DOI:** 10.3390/pathogens14121266

**Published:** 2025-12-10

**Authors:** Zhenyue Li, Jun Dai, Zitong Yang, Mingda Zhang, Xuemeng Wang, Chenchen Ge, Yi Lu, Wenhao Feng, Sihui Song, Cheng Zhang, Huan Cui, Zhendong Guo

**Affiliations:** 1 Changchun Veterinary Research Institute, Chinese Academy of Agriculture Sciences, Changchun 130122, China; 2 College of Veterinary Medicine, Hebei Agricultural University, Baoding 071000, China; 3 Laboratory of Science and Education Department, Baoding Hospital, Beijing Children’s Hospital Affiliated to Capital Medical University, Baoding 071000, China

**Keywords:** Ebola virus (EBOV), isothermal amplification, rapid diagnosis, recombinase-aided amplification (RAA), visual detection

## Abstract

Ebola virus (EBOV) infection constitutes a significant global public health threat, and no curative treatment is currently available for it. Rapid and accurate detection of EBOV nucleic acid is crucial for controlling the spread of Ebola virus disease (EVD). The gold standard for EBOV diagnosis is real-time reverse transcription polymerase chain reaction (RT-qPCR), which requires costly equipment and skilled personnel, potentially hindering its application for rapid detection, especially in resource-limited settings. Consequently, there is an urgent need to develop a simple, accurate, and rapid diagnostic method for EVD. In this study, a real-time reverse transcription recombinase-aided amplification (RT-RAA) assay was developed for the specific visual detection of the conserved region of the EBOV nucleoprotein (NP) gene. The RT-RAA assay can be completed within 30 min at 42 °C, and results can be visualized using a portable blue light imager. The assay exhibited strong analytical specificity toward EBOV. No cross-reactivity was observed with any of the other public-health-relevant viruses tested. The visual RT-RAA assay demonstrated sensitivity comparable to RT-qPCR, detecting 52 copies per reaction at a 95% probability level, whereas RT-qPCR required 74 copies per reaction. The RAA method demonstrated excellent repeatability and stability, with intra-assay and inter-assay CVs less than 5% and 7%, respectively. These results clearly indicate that the visual RT-RAA method is specific, accurate, simple, rapid, and reliable for EBOV detection.

## 1. Introduction

Ebola virus disease (EVD), formerly Ebola hemorrhagic fever (EHF), is a severe and often fatal illness in humans and primates [1]. Ebola virus (EBOV) is a single-stranded, negative-sense RNA virus from the order *Mononegavirales*, the Filoviridae family, genus Ebolavirus, with an 18.9 kb genome encoding genes such as NP, VP35, VP40, GP, VP30, VP24, and L [2]. EBOV, which highly pathogenic and has a mortality rate of 50% to 90%, spreads primarily through contact with the blood, secretions, or body fluids of an infected person [3]. The symptoms of EVD are very similar to those of Marburg virus, including nausea, vomiting, diarrhea, general soreness, internal bleeding, external bleeding, fever, etc. [4]. EBOV can affect people of virtually any age, with no sex or race predilection [5,6].

Given the severe characteristics of EVD, accurate and rapid diagnosis is crucial, but current diagnostic methods have limitations. Early diagnosis and isolation can help reduce virus transmission, which increases the demand for accurate, rapid and sensitive laboratory testing methods. The diagnostic methods used include viral nucleic acid detection, such as RT-PCR, loop-mediated isothermal amplification (LAMP) or enzyme-linked immunosorbent assay (ELISA) [7,8,9]. RT-PCR is time-consuming, costly, and it requires skilled technicians, which limits its use in low-resource settings. ELISAs require high virus levels, which have low sensitivity for low-titer samples and hinder early diagnosis. LAMP, though sensitive, has high false-positive rates and contamination issues. The colloidal gold method is user friendly, instrument free, and adaptable to various conditions. The colloidal gold method, which typically relies on antibody–antigen recognition on a lateral flow immunoassay (LFIA) strip using gold nanoparticles as visible labels [10]. Furthermore, it skips nucleic acid extraction, completing detection in minutes. The colloidal gold method is suitable for widespread use in resource-limited areas but has poor sensitivity and may lead to false-negative results. Therefore, an accurate and rapid EVD detection method is needed.

To overcome these limitations, novel diagnostic techniques such as recombinase-aid amplification (RAA) have been developed. RAA is a novel isothermal amplification method in vitro that involves reactions lasting 15–30 min at 37–42 °C using recombinase, polymerase and single-stranded DNA binding proteins (SSB) [11]. A specific fluorescent probe enables real-time DNA detection, whereas the addition of reverse transcriptase allows real-time monitoring of RNA amplification [12,13]. Moreover, the products can be visualized with a portable blue-light imager [14,15]. There are genomic differences among Ebola virus (EBOV) subtypes. The nucleoprotein (NP) gene is one of the regions with the highest conservation across the entire EBOV genome. This high conservation ensures the specificity of detection and the applicability across different EBOV strains, serving as a core prerequisite for diagnostic targets. In contrast, the glycoprotein (GP) gene exhibits a high mutation rate, which easily leads to missed diagnoses in detection due to mutations. Although the polymerase (L) gene is relatively conserved, it has a length of 7.5 kb, much longer than that of the NP gene (approximately 1.8 kb). Additionally, the L gene has low continuity and concentration of conserved regions, making it difficult to design ideal detection targets. Furthermore, among the 8 structural genes of EBOV, the NP gene has the highest copy number, which can enhance the sensitivity of reverse transcription-recombinase aided amplification (RT-RAA) detection.

In this study, we developed a real-time RT-RAA method for rapid, accurate and visible EBOV detection. Owing to its advantages of rapidity, accuracy, strong specificity and easy operation, RAA is ideal for field detection. Moreover, when a portable blue light imager (excitation wavelength of 480 nm) is used, the amplified products can be observed visually, making it suitable for emergencies and resource-limited settings.

## 2. Materials and Methods

### 2.1. Virus Strains and Plasmid

Total RNA was stored in RNAstable^®^ (Biomatrica, San Diego, CA, USA) [16]. The following viruses were used and maintained in our laboratory: influenza A viruses (A/Sichuan/SC99/2019 (H1N1), A/Hebei/BD79/2018 (H3N2), A/goose/Hebei/GD07/2019 (H5N6), A/environment/Hebei/621/2019 (H7N9), A/Fujiansiming/19/2021 (H9N2)), influenza B viruses (Yamagata and Victoria), respiratory syncytial viruses (A and B), and the EBOV-NP plasmid (pMD18-T-NP, GenBank: AY142960).

### 2.2. Nucleic Acid Extraction

TRIzol reagent (Magen, Guangzhou, China) was used to extract the viral RNAs of H1N1, H3N2, H5N6, H7N9, H9N2, RSV-A, RSV-B, IBV-Y, and IBV-V. All the extracted RNA was stored at −80 °C until use. pMD18-T-NP plasmid extraction was performed with a TIANprep Mini Plasmid Kit (Tiangen, Beijing, China). The extracted plasmids were preserved at −20 °C until they were needed. DNA copy number was calculated using the following formula: DNA copy number/μL = [plasmid concentration (ng/μL) × 10^−9^ × 6.02× 10^23^]/[DNA length (nt) × 660].

### 2.3. Design of Primers and Exo Probes

To design a robust RT-RAA assay targeting the Ebola virus (EBOV) nucleoprotein (NP) gene, we first collected representative NP gene sequences from multiple EBOV species and strains. All sequences used for alignment and conserved-region selection are listed in Supplementary Table S1, including virus species, strain names, years of isolation, and GenBank accession numbers.

A multiple-sequence alignment was performed using DNAStar MegAlign Pro (version 17.6) with the ClustalW algorithm under default parameters. This alignment enabled us to compare NP gene sequences from *Zaire ebolavirus*, *Sudan ebolavirus*, *Bundibugyo ebolavirus*, *Taï Forest ebolavirus*, and *Bombali ebolavirus* strains. Regions showing high nucleotide identity across the *Zaire ebolavirus* strains and relatively conserved patterns across other EBOV species were further examined as potential candidates for primer binding [15].

To ensure optimal primer performance, candidate regions were evaluated according to the following criteria: (1) high sequence conservation, avoiding variable sites identified through multiple alignment; (2) moderate GC content (40–60%) to ensure stable yet efficient annealing; (3) minimal predicted secondary structures, assessed using OligoAnalyzer 3.1 (Integrated DNA Technologies, Coralville, IA, USA); (4) absence of repetitive motifs or long homopolymers that may impair recombinase-aided amplification; (5) suitable amplicon length (80–180 bp) compatible with RAA reaction kinetics.

Based on these criteria, a highly conserved region located within the central portion of the NP gene was selected as the amplification target. Final primers and exo-probe were designed within this region using Primer Premier 5.0, and were screened to avoid dimer formation, cross-dimers, or hairpin structures.

Briefly, randomly selected forward primers were used to select the best reverse primers, which were then used to select all the forward primers. Multiple primer screening was required to find more sensitive primer pairs. Finally, a good primer pair was identified. The primers and probes of the NP-based RT-qPCR assay for EBOV were designed based on a previously reported study [17] and are listed in Table 1. The above primers and probes were synthesized by Comate Biotech Co., Ltd. (Changchun, China).

### 2.4. Real-Time RT-RAA Assays and Visual Detection

**Figure 1:**
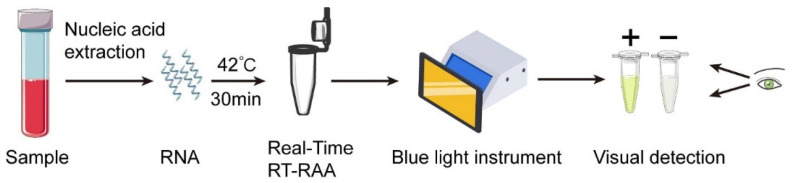
Schematic diagram of the visual RT-RAA assay for EBOV. The entire RT-RAA reaction can be finished in 30 min at 42 °C, and the results can be observed visually through a portable blue light imager (excitation wavelength of 480 nm).

Real-time RT-RAA kits (#WLRE8208KIT) were purchased from Amp-Future Biotech Co., Ltd. (Weifang, China). In brief, the reaction was performed in a total volume of 25 μL containing: 14.7 μL of Buffer A; 4.75 μL of nuclease-free water; 0.3 μL of exo probe (10 μM); 1.0 μL of forward primer (10 μM); 1.0 μL of reverse primer (10 μM); 2.0 μL of nucleic acid template; and 1.25 μL of Buffer B. The reaction tube was placed in a 7500 real-time PCR system (42 °C, 30 min, 1 cycle per minute) to monitor the fluorescence signal. Fluorescence signals during RT-RAA were monitored using an ABI 7500 Real-Time PCR System at 42 °C for 30 min, with fluorescence acquisition set to one cycle per minute. Data were collected using the ABI 7500 System SDS Software v2.0.6, and fluorescence was recorded on the FAM channel (excitation 495 nm, emission 520 nm). In addition, a portable blue light imager (excitation wavelength of 480 nm) (TGreen, China Tiangen Biotechnology Co., Ltd., Beijing, China) was used for visual detection (Figure 1). Although fluorescence monitoring was performed using an ABI 7500 real-time PCR system, the RT-RAA reaction itself only requires a constant 42 °C incubation. In parallel tests, the same 30 min incubation was reproducibly achieved using a low-cost constant-temperature metal heating block and a portable battery-powered incubator. Therefore, the amplification step does not rely on laboratory-grade thermocyclers.

### 2.5. RT-qPCR Assay

The RT-qPCR volume (25 μL) for EBOV included 12.5 μL of 2× One Step PrimeScript III RT-qPCR Mix; 9 μL of nuclease-free water; 0.5 μL of 10 μM forward primer, reverse primer or probe; and 2.0 μL of nucleic acid template. The reaction mixture was placed in a 7500 real-time PCR system (Applied Biosystems, Foster City, CA, USA). The RT-qPCR program was as follows: 52 °C for 5 min, 95 °C for 10 s, then 40 cycles at 95 °C for 5 s and 60 °C for 30 s.

### 2.6. Optimizing the Total Volume of Visual RT-RAA Reaction

To determine the effect of the total reaction volume on the amplification efficiency, reactions with volumes of 15, 20, 25, 30, 35, 40, 45 and 50 μL were tested using visual real-time RT-RAA using the best primer pair.

### 2.7. Test of the Specificity of Visual RT-RAA Reactions

The specificity of EBOV visual RT-RAA assays was assessed by using other viruses, including H1N1, H3N2, H5N6, H7N9, H9N2, IBV-Y, IBV-V, RSV-A, and RSV-B. In addition to conserved-region selection, an in silico cross-reactivity analysis was performed to evaluate the theoretical specificity of the EBOV NP primers and probe. Using NCBI Primer-BLAST and multiple-sequence alignment (MegAlign Pro, DNAStar, version 17.6), the primer–probe set was compared against representative NP gene sequences from major filoviruses and hemorrhagic-fever pathogens, including Sudan ebolavirus (SUDV), Bundibugyo ebolavirus (BDBV), Taï Forest ebolavirus (TAFV), Bombali ebolavirus (BOMV), Reston ebolavirus (RESTV), Marburg virus (MARV), and Lassa virus (LASV).

The predicted mismatches for each virus species and the likelihood of off-target amplification are summarized in Supplementary Table S2. These analyses showed that non-Zaire ebolaviruses contain ≥4 mismatches within primer-binding regions, and no significant homology was detected with MARV or LASV sequences, indicating high theoretical specificity of the assay.

To evaluate the nonspecific amplification risk associated with RT-RAA, repeatability and reproducibility tests were performed using non-template controls (NTCs) and off-target viral nucleic acids. Eight independent NTC reactions were prepared using nuclease-free water as template. To assess off-target amplification, eight replicates of each non-EBOV viral RNA template (H1N1, H3N2, RSV-A, RSV-B) were tested under identical RT-RAA reaction conditions. Fluorescence signals were recorded throughout the amplification process, and baseline readings, standard deviation (SD), and coefficient of variation (CV%) were calculated to determine the stability of negative reactions.

### 2.8. Sensitivity Test of Visual RT-RAA Reactions

The visual RT-RAA assay was conducted under optimal reaction conditions. Tenfold serial dilutions of pMD18-T-NP at concentrations ranging from 1 × 10^6^ to 1 × 10^0^ copies/2 μL were obtained. Each 2 μL dilution was used as a template for real-time RT-RAA to assess the sensitivity of visual detection. The same templates were detected in parallel using RT-qPCR assays. To analyze the amplification limit more accurately, the dilution series (1 × 10^6^–1 × 10^0^ copies per reaction) was used as a template for 8 independent runs in both assays, and IBM’s Statistical Products and Services solution (SPSS) software (version 29.0) was used to analyze the data through probit regression.

### 2.9. Analysis of Repeatability and Stability

To evaluate the intragroup and intergroup reproducibility of the RT-RAA method, high, medium, and low concentrations of EBOV pMD18-T-NP plasmids (10^6^, 10^4^, and 10^2^ copies per reaction) were used for repeated detection in a single run or three independent runs at different times, and the coefficient of variation (CV) of the threshold time was calculated.

### 2.10. Statistical Analysis

SPSS software from IBM was used to perform probit regression analysis at the 95% probability level to determine the amplification limit.

## 3. Results

### 3.1. Optimization of the Best Primers for Visual RT-RAA Assay

**Figure 2:**
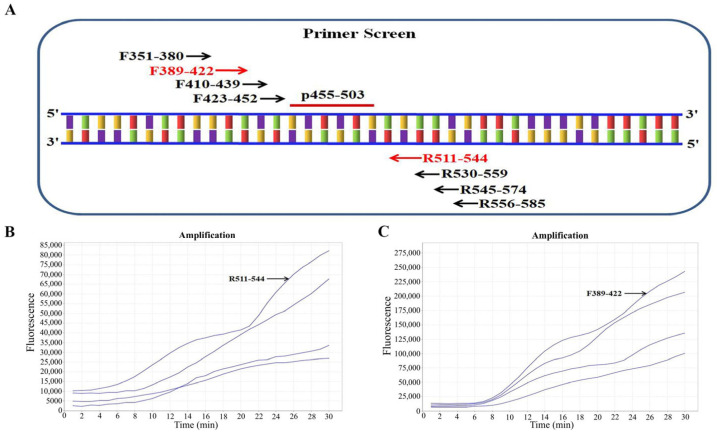
Screening of the best primer pair. (**A**) Schematic view of primer screening. The numbers in the primer names indicate the location within the NP gene of Zaire ebolavirus isolate IRF0164 (GenBank accession no. KY425656.1). (**B**) Results of reverse primer screening. F351-380 was randomly selected as the forward primer to screen all four reverse primers. (**C**) Screening results of the forward primer. The reverse primer R511-544 was used for screening all four forward primers.

The multiple-sequence alignment further revealed that the central portion of the NP gene (approximately nt 450–580) exhibited the highest pairwise identity (>97%) among all representative Zaire ebolavirus strains, including Mayinga, Kikwit, and Makona lineages. No mutation hotspots or clusters of ≥3 consecutive mismatches were identified in this segment. This high conservation, combined with its moderate GC content (41–48%) and minimal predicted secondary structures, supported the selection of this region as the optimal target for primer and probe design. First, we chose an ideal probe (p455-503) (Table 1). Four forward (F351-380, F389-422, F410-439, and F423-452) and four reverse (R511-544, R530-559, R545-574, and R556-585) candidate primers were subsequently designed around the target region of the probe p455-503 (Figure 2A). Strategies for screening primer pairs were based on previous methods [15]. In brief, we first screened all four reverse primers with the randomly selected forward primer F351-380 and found R511-544 to have the best amplification (Figure 2B). Using R511-544 as the fixed reverse primer, we then screened all four forward primers and confirmed F389-422 to exhibit the best amplification performance (Figure 2C). Thus, the primer pair F389-422/R511-544 was the best.

### 3.2. Optimizing the Total Volume of the Visual RT-RAA Assay

**Figure 3:**
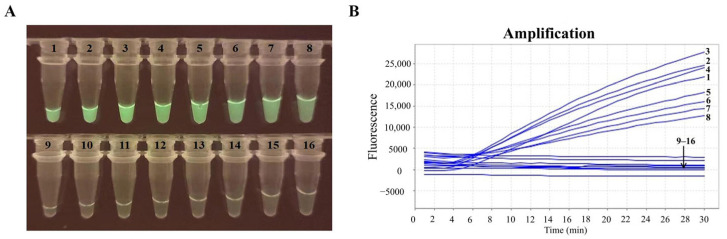
Optimization of total reaction volume for visual RT-RAA detection. (**A**) Results of visual RT-RAA detection with a portable blue light imager. (**B**) Results of the real-time RT-RAA assay through real-time fluorescence readout. Tubes or curves 1–8 (positive sample group) and 9–16 (negative control group) represent total reaction volumes of 15, 20, 25, 30, 35, 40, 45, and 50 μL, respectively. *n* = 3 independent runs.

The optimal total reaction volume was determined to be 25 μL, as this volume yielded the strongest and brightest fluorescence signals (Figure 3). Therefore, this volume was identified as the optimal total reaction volume for all subsequent visual RT-RAA detection assays.

### 3.3. Test of the Specificity of Visual RT-RAA Reactions

**Figure 4:**
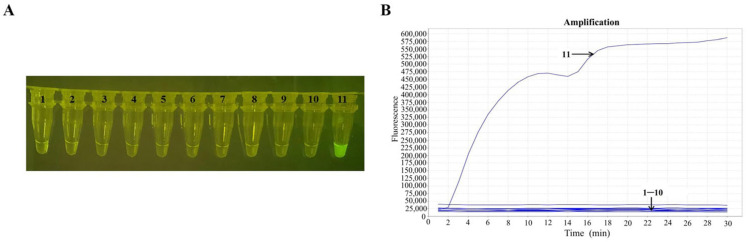
Specificity test of the visual RT-RAA reaction for EBOV. (**A**) Results of the visual RT-RAA assay through a portable blue light imager. (**B**) Results of the RT-RAA assay with fluorescence readout. Tubes or curves 1–11 represent H1N1, H3N2, H5N6, H7N9, H9N2, IBV-Y, IBV-V, RSV-A, RSV-B, the negative control (no template control), and EBOV, respectively. *n* = 3 independent runs.

The visual RT-RAA assay showed positive results for EBOV and no cross- reactivity with H1N1, H3N2, H5N6, H7N9, H9N2, IBV-Y, IBV-V, RSV-A, RSV-B, or the negative control (Figure 4A,B). The visual RT-RAA assay showed high analytical specificity against the respiratory viruses available in our laboratory. To further assess theoretical cross-reactivity, we performed an in silico analysis using NP sequences from major filoviruses and hemorrhagic-fever viruses (SUDV, BDBV, TAFV, BOMV, RESTV, MARV, LASV). The EBOV NP primer–probe set demonstrated ≥ 4 mismatches for non-Zaire ebolaviruses and no detectable homology with MARV or LASV (Supplementary Table S2), supporting the assay’s theoretical specificity. To further validate the specificity of the RT-RAA assay, the repeatability and reproducibility of the NTCs and off-target controls were evaluated. Eight independent NTC reactions produced no detectable amplification, with baseline fluorescence values showing minimal variation (CV < 3%). Similarly, eight replicates of off-target reactions using H1N1, H3N2, RSV-A, and RSV-B RNA remained negative in all runs without any signal drift or nonspecific amplification. These results demonstrate high stability of the negative reactions and support the specificity of the assay (Supplementary Table S3). This finding indicated that the developed visual RT-RAA assay was thus highly specific for EBOV.

### 3.4. Sensitivity Test of Visual RT-RAA Reactions

**Figure 5:**
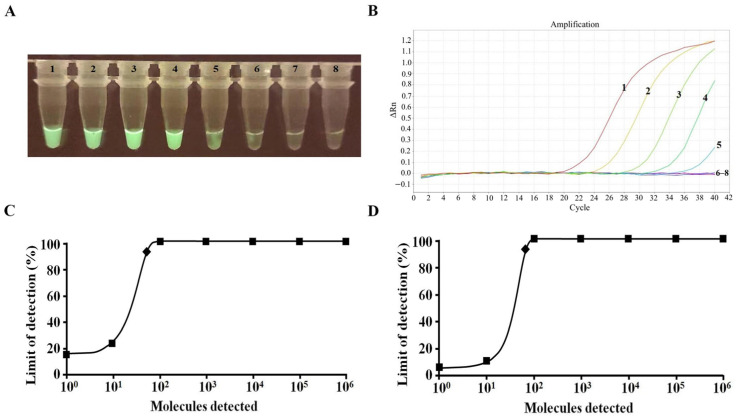
Sensitivity test for EBOV. (**A**) Results of visual RT-RAA detection through a portable blue light imager. Tubes 1–8 correspond to 10^6^–10^0^ copies and negative control, respectively. (**B**) Results of RT-qPCR. Curves 1–8 correspond to 10^6^–10^0^ copies and negative control, respectively. (**C**) A rhomboid was used to mark the detection limit of the visual RT-RAA reaction at 95% probability (52 copies/reaction). (**D**) A rhomboid was used to mark the detection limit of the RT-qPCR at 95% probability (74 copies/reaction). *n* = 8 independent runs.

Sensitivity analysis demonstrated that the amplification limit of the visual RT-RAA assay was 10 copies/reaction (Figure 5A). The RT-qPCR results indicated that the amplification limit of the RT-qPCR assay was 100 copies/reaction (Figure 5B). Probit regression analyses revealed that the two assays detected a detection limit of 52 and 74 copies/reaction, respectively, with 95% reliability (Figure 5C,D). The visual RT-RAA assay produced detectable fluorescence at approximately 10 copies/µL; however, statistical evaluation using probit regression demonstrated a 95% detection limit of 52 copies/reaction. Therefore, the final analytical sensitivity of the assay is defined as 52 copies/reaction (95% probability), while 10 copies/µL represents only the visual detection threshold.

### 3.5. Repeatability and Stability Analysis

To evaluate the repeatability and stability of the RT-RAA method, it was applied to detect EBOV pMD18-T-NP plasmids at concentrations of 10^6^, 10^4^, and 10^2^ copies/reaction. These tests were repeated three times in parallel. The intra-assay CVs for high (10^6^), medium (10^4^), and low (10^2^) concentrations were 4.92%, 1.61%, and 2.06%, respectively, while the inter-assay CVs for the same concentration gradients were 6.64%, 4.99%, and 3.65%, respectively. Both the intra-assay CVs (≤5%) and inter-assay CVs (≤7%) were within the acceptable range, which demonstrates that the RT-RAA method has excellent repeatability and stability (Table 2).

## 4. Discussion

Owing to the extremely high pathogenicity and mortality of EVD, the World Health Organization (WHO) listed the 2014 West Africa and 2019 Congo Ebola outbreak as “Public Health Emergency of International Concern (PHEIC)”. The EBOV, which has a biosafety level of 4, requires strict protective measures. EVD caused by the EBOV is characterized by short latency, acute onset, high infectivity and high mortality. At present, although preventive vaccines or specific therapeutic drugs for EVD have been developed, early diagnosis is very important for the effective prevention and control of EVD. Therefore, the development of rapid and accurate EBOV detection methods is crucial.

Currently, methods employed in laboratories for the detection of EBOV primarily encompass virus isolation and identification, nucleic acid detection, antigen–antibody detection, etc. Virus isolation and identification constitute the gold standard for disease diagnosis; however, this method usually demands advanced laboratory conditions and skilled operation and is time-consuming, with biosecurity risks. The nucleic acid detection method has emerged as a crucial method. According to whether nucleic acid detection depends on the temperature cycle, nucleic acid detection can be categorized into variable-temperature amplification and constant-temperature amplification. Variable temperature amplification methods, such as PCR, are time-consuming and require precise instruments. In contrast, isothermal amplification methods, such as LAMP, react at a constant temperature; are fast, sensitive, and efficient; and require less equipment. These methods are very suitable for onsite detection in resource-limited environments. However, LAMP is prone to produce false positive results, which limits its broader application. Currently, the main application of antigen–antibody detection is ELISA detection technology. Although this detection method is relatively mature, compared with nucleic acid detection technology, the process is still more cumbersome and time-consuming. However, current virus laboratory methods for the rapid detection of sudden infectious diseases are limited. These existing methods hinder their application in resource-limited or emergency scenarios, highlighting the need for a more optimal detection approach.

The RT-RAA assay is distinguished by its rapid detection, with results available within 30 min at a constant temperature of 42 °C. This is a marked improvement over RT-qPCR, which necessitates complex thermal cycling and longer processing times [18,19]. The swift turnaround of the RT-RAA assay is crucial in outbreak situations, where timely diagnosis and intervention can curb the spread of the virus and reduce mortality rates. A key advantage of the RT-RAA assay is its simplicity and ease of use. It requires minimal equipment and can be performed by personnel with limited training, making it particularly suitable for field conditions and low-resource settings [20]. The use of a portable blue light imager for visual detection further enhances its practicality in remote areas, where access to advanced laboratory infrastructure may be restricted [21].

In this study, we developed a visual RT-RAA method for EBOV detection that is rapid, accurate, and specific. The amplification products can be visualized by a portable blue light imager (excitation wavelength of 480 nm). This method is ideal for EBOV detection in settings with limited medical resources and emergencies. The assay was positive for EBOV and had no cross-reactivity with other important viruses. Sensitivity analysis revealed that visual RT-RAA (52 recombinant plasmid copies/reaction at 95% reliability) was equivalent to the RT-qPCR assay (74 recombinant plasmid copies/reaction at 95% reliability) reported in the literature. This high sensitivity is essential for early diagnosis and effective containment of the virus. The RAA method was employed to assess repeatability and stability by detecting three concentrations of EBOV pMD18-T-NP plasmids. The RAA method exhibited excellent repeatability and stability, with low intra-assay (<5%) and inter-assay (<7%) CVs. Consistency in results is critical for the reliability of a diagnostic method, particularly in clinical and field settings where decisions based on test results have significant consequences. It is suitable for initial screening of EBOV in epidemic areas and regions with limited resources, indicating its potential for early diagnosis, point-of-care testing (POCT), and quarantine applications. Although this study evaluated specificity using laboratory-available respiratory viruses, EBOV diagnostics also require evaluation in human background RNA and against blood-borne hemorrhagic-fever pathogens. Due to biosafety limitations, such experiments could not be performed. Instead, we conducted an in silico analysis (Supplementary Table S2), which showed low theoretical cross-reactivity with other filoviruses and no homology with MARV or LASV. Future studies will incorporate human plasma matrices, in vitro-transcribed EBOV NP RNA, or synthetic gBlock controls to establish clinically relevant specificity.

The development of the RT-RAA assay represents a significant advancement in rapid EBOV diagnostics. Its application in outbreak scenarios and resource-limited environments can play a crucial role in controlling EVD. Future research should explore the broader application of the RT-RAA assay in detecting other infectious diseases, potentially establishing it as a universal platform for pathogen detection. Large-scale validation studies are needed to confirm the assay’s efficacy and reliability in diverse real-world settings. Integrating RT-RAA with other diagnostic technologies, such as CRISPR-based detection systems, could further enhance its sensitivity and versatility. For instance, combining RT-RAA’s rapid amplification with CRISPR’s high specificity could enable more accurate and efficient detection of EBOV in complex samples. CRISPR technology has already shown promise in increasing the sensitivity of virus detection, including for EBOV [22]. This study has several important limitations. First, the analytical evaluation was conducted primarily using EBOV NP plasmid templates and nucleic acids from laboratory-maintained respiratory viruses, rather than authentic or inactivated EBOV-positive clinical material. Validation with live or inactivated EBOV could not be performed because our institution does not have access to BSL-4 facilities. Second, the experimental specificity panel did not include other filoviruses such as SUDV, BDBV, TAFV, or BOMV; specificity for these species is therefore based solely on in silico mismatch analysis. Third, although the RT-RAA reaction operates at 42 °C, real field-condition incubation using truly portable heating devices was not evaluated in this study. These limitations highlight that the present work represents analytical development, and further validation under BSL-4 and field-relevant conditions will be required before operational deployment.

It should be noted that although EBOV is an RNA virus, the analytical evaluation of this assay was performed using plasmid DNA. Previous studies indicate that the reverse-transcription step in RT-RAA reactions has minimal influence on amplification kinetics, typically altering threshold times by less than 1–2 min compared with DNA-based RAA reactions. Therefore, plasmid DNA is suitable for initial analytical validation. Nevertheless, further evaluation using in vitro-transcribed EBOV NP RNA or authentic EBOV RNA is required to fully demonstrate the assay’s RT-RAA performance for real-world applications. Due to biosafety constraints, EBOV-positive RNA could not be obtained. Our institute does not have access to a BSL-4 laboratory, and there are no locally reported EVD cases in China. Therefore, plasmid DNA containing the EBOV NP gene (pMD18-T-NP) was used solely for analytical evaluation of the RT-RAA assay. This approach enables preliminary assessment of amplification performance but does not replace validation using RNA templates, which will be addressed in future work.

## 5. Conclusions

This study successfully developed a RT-RAA assay for the detection of EBOV, specifically targeting the NP gene. The primary objective was to create a diagnostic tool that is rapid, accurate, and feasible for use in resource-limited settings and emergency situations. The RT-RAA method demonstrated several key advantages over existing diagnostic techniques, making it a valuable addition to the field of infectious disease detection. To evaluate its practicality, we systematically assessed the assay’s performance characteristics. In this study, the F389-422/R511-544 primer set was identified through multiple rounds of primer screening. This set targets conserved regions of the NP gene across major EBOV subtypes (including Zaire and Sudan strains), addressing the limitation of insufficient detection specificity of universal RT-RAA kits when applied to highly variable viruses. Meanwhile, validation of plasmid standards in this study aimed to establish basic performance metrics of the method, such as limit of detection and repeatability. Notably, the innovation of this study resides in the precise alignment between method performance and on-site scenario demands, rather than mere innovation in technical principles. It is worth noting that the slight difference between the analytical LoDs of RT-RAA (52 copies/reaction) and RT-qPCR (74 copies/reaction) is within the expected range of assay variability. The RT-qPCR assay used in this study employed a commercial, non-EBOV-optimized kit, which may partly explain the modestly weaker performance compared with RT-RAA. Because pure plasmid DNA was used for all LoD evaluations, no matrix inhibition effects were present. Therefore, the small numerical difference should not be interpreted as a true sensitivity advantage of RT-RAA over RT-qPCR.

The RT-RAA assay offers several distinct advantages. First and foremost, it is a rapid diagnostic method, capable of detecting EBOV within 30 min at a constant temperature of 42 °C. This is significantly faster than the traditional RT-qPCR, which requires complex thermal cycling and longer processing times, and access to specialized laboratory equipment. The rapid turnaround time of the RT-RAA assay is crucial for timely decision-making in outbreak scenarios, where timely intervention is critical. Another significant advantage of the RT-RAA assay is its simplicity and ease of use. The assay can be performed with minimal equipment and does not require highly trained personnel. This makes it particularly suitable for field conditions and low-resource settings where access to advanced laboratory infrastructure and skilled technicians may be limited. The ability to perform the assay using a portable blue light imager further enhances its utility in remote and resource-constrained environments. The specificity of the RT-RAA assay was rigorously evaluated, and the results demonstrated that the assay is highly specific for EBOV. There was no cross-reactivity with other significant viruses, including various strains of influenza and respiratory syncytial viruses. This high specificity is critical for avoiding false-positive results, which can lead to unnecessary alarm and resource allocation, during an outbreak. In terms of sensitivity, the RT-RAA assay was found to be comparable to gold standard RT-qPCR method. The assay could detect as few as 52 copies of EBOV per reaction with 95% probability, which is on par with the sensitivity of RT-qPCR (74 copies per reaction with 95% probability). The ability to detect low viral loads is essential for two key goals: early diagnosis and effective containment of the virus. Additionally, the repeatability and stability of this RT-RAA assay was thoroughly assessed. The assay showed excellent repeatability, with intra-assay and inter-assay CV values being less than 5% and 7%, respectively. This indicates that the assay produces consistent and reliable results, which is crucial for its implementation in clinical and field settings. Overall, the development of the RT-RAA assay represents a significant advancement in the field of rapid diagnostic testing for EBOV. Its speed, simplicity, specificity, and sensitivity make it a powerful tool for early detection and outbreak management. It should be noted that all analytical evaluations in this study were conducted under laboratory conditions. While the RT-RAA reaction can be performed using simple 42 °C heating devices, true field deployment will require additional developments such as lyophilized reagents, extraction-free sample processing, and fully portable visual-readout systems. Thus, this assay should currently be considered “potentially field-deployable,” pending further engineering optimization.

Future research should focus on the broader application of the RT-RAA assay for other infectious diseases, exploring its potential as a universal platform for rapid and accurate pathogen detection. Additionally, field trials and large-scale validation studies are needed to confirm the assay’s efficacy and reliability in diverse real-world settings. Integrating the RT-RAA assay with other diagnostic technologies, such as CRISPR-based detection systems, could further enhance its sensitivity and versatility. However, additional developments such as reagent lyophilization, extraction-free sample processing, and visual readout formats will be necessary to achieve true field deployment. In conclusion, this RT-RAA assay provides a valuable and effective solution for the rapid detection of EBOV. Its deployment in field conditions and resource-limited settings can significantly improve the management of EVD outbreaks, ultimately contributing to global health security and pandemic preparedness. The ongoing advancements in EBOV research and diagnostic technologies underscore the importance of innovative strategies in the fight against infectious diseases.

## Figures and Tables

**Table 1:** Information on the primers and probes used in visual RT-RAA and RT-qPCR detection of EBOV (Amplicon length = 129 bp).

Primers/Probes	Sequences (5′→3′)	GC (%)	Position ^a^	Source
F389-422	TTAAGAGAACACTTGCTGCCATGCCGGAAGAGGA	45.8	389–422	This study
R511-544	GCTCTGCATGTACTTGAATTTGCCTTTGAACCTT	41.7	511–544	This study
P455-503	CCTTTGCAAGTCTATTCCTTCCGAAATTGG(FAM-dT) A(THF)(BHQ1-dT) AGGAGAAAAGGCTTG[C3-spacer]	55.0	455–503	This study
F	GCAGAGCAAGGACTGATTCA	-	538–557	[17]
R	GTTCGCATCAAACGGAAAAT	-	598–617	[17]
Probe	FAM-CAACAGCTTGGCAATCAGTTGGACA-TAMRA	-	563–587	[17]

^a^ The position of the primers/probes refers to the nucleoprotein gene (NP gene) of Zaire ebolavirus isolate IRF0164 (GenBank accession no. KY425656.1).

**Table 2:** Threshold time (s) and coefficient of variation (CV%) of the RT-RAA assay at different EBOV NP plasmid concentrations (*n* = 3).

Plasmids Concentration	Repeatability (Intra-Batch Assay)			Reproducibility (Inter-Batch Assay)		
	Mean	SD	CV (%)	Mean	SD	CV (%)
High (10^6^)	102.33	5.03	4.92	105.67	7.02	6.64
Medium (10^4^)	218.67	3.51	1.61	223.67	11.15	4.99
Low (10^2^)	412.33	8.50	2.06	416.33	15.18	3.65

Mean: average threshold time (seconds) of three independent real-time RAA reactions; SD: standard deviation; CV: coefficient of variation.

## Data Availability

All data supporting the findings of this study are included within the article. Additional data are available from the corresponding author upon reasonable request.

## Supplementary Materials

The following supporting information can be downloaded at: https://www.mdpi.com/article/10.3390/pathogens14121266/s1, Table S1: EBOV NP gene sequences used for multiple-sequence alignment; Table S2: In-silico cross-reactivity analysis of EBOV NP RT-RAA primers and probe against filoviruses and other hemorrhagic-fever viruses; Table S3: Repeatability and reproducibility of NTC and off-target controls (n = 8).
