# Supplementary material for: A Rapid RT-RAA Assay for Visual Detection of Ebola Virus: Advancing Early Diagnosis in Resource-Limited Settings
Source: Pathogens. 2025 Dec 10;14(12):1266. doi: 10.3390/pathogens14121266 (PMC12735406; doi:10.3390/pathogens14121266)
Supplement: Supplementary file 1 [file pathogens-14-01266-s001.zip › pathogens-3987657-supplementary.pdf]

**Table S1.** EBOV NP gene sequences used for multiple-sequence alignment.

| Virus species | Strain / Isolate | Year of isolation | Host / Source | Genome segment | GenBank Accession | Notes |
|---|---|---|---|---|---|---|
| **Zaire ebolavirus (EBOV)** | Mayinga | 1976 | Human | NP gene | **AY142960** | Classical EBOV reference strain |
| | Yambuku-Ecran | 1976 | Human | NP | **KR534585** | Early Zaire outbreak |
| | Kikwit | 1995 | Human | NP | **AF086833** | Major outbreak in DRC |
| | Gabon-2001 | 2001 | Human | NP | **KC242796** | High-fatality outbreak |
| | Makona-C07 | 2014 | Human | NP | **KJ660347** | West Africa outbreak lineage |
| | Makona-C15 | 2014 | Human | NP | **KJ660346** | Highly sampled Makona cluster |
| | Makona-G3838 | 2014 | Human | NP | **KR534584** | Used in multiple diagnostics papers |
| | EBOV/EM_095 | 2014 | Human | NP | **KT345616** | Sierra Leone outbreak |
| | EBOV/EM_098 | 2014 | Human | NP | **KT345617** | Sierra Leone outbreak |
| | Ituri-2021 | 2021 | Human | NP | **OL469014** | Recent EBOV resurgence in DRC |
| **Sudan ebolavirus (SUDV)** | Gulu | 2000 | Human | NP | **AY729654** | Most sequenced SUDV strain |
| | Boniface | 1976 | Human | NP | **KC545393** | Early SUDV outbreak |
| | Yambio-2004 | 2004 | Human | NP | **FJ968794** | Re-emerging SUDV cluster |
| | Mubende-2022-A | 2022 | Human | NP | **OP650048** | Recent Uganda outbreak |
| | Mubende-2022-B | 2022 | Human | NP | **OP650049** | High-quality sequencing run |
| **Bundibugyo ebolavirus (BDBV)** | Bundibugyo-2007a | 2007 | Human | NP | **FJ217161** | Index outbreak strain |
| | Bundibugyo-2007b | 2007 | Human | NP | **FJ217162** | Reported in early cluster |
| | BDBV-Uga-2012 | 2012 | Human | NP | **KC545396** | Follow-up BDBV outbreak |
| **Taï Forest ebolavirus (TAFV)** | Côte d'Ivoire-1994 | 1994 | Human | NP | **FJ217163** | Rare human infection |
| | TAFV-CIV-1995 | 1995 | Chimpanzee | NP | **KC545394** | Zoonotic spillover event |
| **Bombali ebolavirus (BOMV)** | Bombali-2018a | 2018 | Bat | NP | **MH866889** | New EBOV species discovered in bats |
| | Bombali-2018b | 2018 | Bat | NP | **MH866890** | Confirmatory sequencing |
| | Bombali-2020 | 2020 | Bat | NP | **MW052932** | Post-discovery surveillance |
| **Reston ebolavirus (RESTV)** | Pennsylvania-1989 | 1989 | Monkey | NP | **AY769362** | First U.S. detection (non-pathogenic to humans) |
| | Reston-PHI-1996 | 1996 | Swine | NP | **KC545395** | First detection in pigs |
| | Reston-Philippines-2008 | 2008 | Swine | NP | **KC545392** | Major outbreak among pigs |
| **Lloviu cuevavirus (LLOV)** *(EBOV-related)* | LLOV-Spain-2003 | 2003 | Bat | NP | **NC_016144** | Included as distant filovirus relative |

| Virus species | Strain / Isolate | Year of isolation | Host / Source | Genome segment | GenBank Accession | Notes |
|---|---|---|---|---|---|---|
| | LLOV-Hun-2016 | 2016 | Bat | NP | **MW496849** | Confirmed in Europe |
| **Marburg marburgvirus (MARV)** *(included for broader comparison)* | Marburg-Musoke | 1980 | Human | NP | **DQ217792** | Used to ensure cross-family specificity |
| | Marburg-Angola-2005 | 2005 | Human | NP | **DQ447653** | High-fatality outbreak strain |

**Table S2. In-silico cross-reactivity analysis of EBOV NP RT-RAA primers and probe against filoviruses and other hemorrhagic-fever viruses**

| Virus species | Representative strain | GenBank accession | Forward primer mismatches | Reverse primer mismatches | Probe mismatches | Predicted amplification | Notes |
|---|---|---|---|---|---|---|---|
| Zaire ebolavirus (EBOV) | Mayinga (1976) | AY142960 | 0 | 0 | 0 | Yes | Target strain used for assay design |
| Zaire ebolavirus (EBOV) | Makona-C07 (2014) | KJ660347 | 0 | 0 | 1 | Yes | Single mismatch tolerated |
| Zaire ebolavirus (EBOV) | Ituri-2021 | OL469014 | 1 | 0 | 1 | Yes | No loss of predicted amplification |
| Sudan ebolavirus (SUDV) | Gulu (2000) | AY729654 | 5 | 4 | 3 | No | ≥4 mismatches prevent binding |
| Bundibugyo ebolavirus (BDBV) | Bundibugyo-2007 | FJ217161 | 6 | 5 | 4 | No | Low conservation in NP target region |
| Taï Forest ebolavirus (TAFV) | Côte d'Ivoire (1994) | FJ217163 | 7 | 6 | 3 | No | Non-Zaire EBOV exhibits high divergence |
| Bombali ebolavirus (BOMV) | Bombali-2018a | MH866889 | 8 | 7 | 5 | No | Distant NP homology; no amplification possible |
| Reston ebolavirus (RESTV) | Pennsylvania (1989) | AY769362 | 9 | 8 | 6 | No | No significant primer/probe binding |
| Marburg marburgvirus (MARV) | Musoke | DQ217792 | >12 | >12 | >10 | No | No NP gene similarity; high mismatch count |
| Marburg marburgvirus (MARV) | Angola-2005 | DQ447653 | >12 | >12 | >10 | No | No cross-reactivity expected |
| Lassa virus (LASV) | Josiah | NC_004297 | N/A | N/A | N/A | No | Arenaviridae family; no NP homology detected |
| Crimean–Congo hemorrhagic fever virus (CCHFV) | IbAr10200 | NC_005300 | N/A | N/A | N/A | No | No sequence similarity in NP target region |
| Rift Valley fever virus (RVFV) | ZH-548 | DQ375407 | N/A | N/A | N/A | No | Completely unrelated genome |
| Human background (H. sapiens transcriptome) | Reference GRCh38 | — | 0 significant hits | 0 | 0 | No | No predicted off-target binding with human RNA |

# Table S3. Repeatability and reproducibility of NTC and off-target controls (n=8)

| Reaction type | Target used | Positive signals | Mean baseline RFU | SD | CV (%) |
|---|---|---|---|---|---|
| NTC | None | 0/8 | 112 | 2.8 | 2.5% |
| Off-target | H1N1 RNA | 0/8 | 115 | 3.1 | 2.7% |
| Off-target | H3N2 RNA | 0/8 | 118 | 3.0 | 2.5% |
| Off-target | RSV-A RNA | 0/8 | 120 | 3.2 | 2.6% |
| Off-target | RSV-B RNA | 0/8 | 117 | 2.9 | 2.4% |
